# Remembering routes: confronting spatial behaviours and sketch maps in individual and collective contexts

**DOI:** 10.3389/fpsyg.2025.1541363

**Published:** 2025-05-02

**Authors:** Teriitutea Quesnot, Bernard Guelton

**Affiliations:** ^1^Département de Géographie, Université de Bretagne Occidentale, Brest, France; ^2^Institut Universitaire de France, Paris, France; ^3^École des arts de la Sorbonne, Université Paris 1 Panthéon-Sorbonne, Paris, France

**Keywords:** collective interactions, landmarks, navigation, sketch maps, spatial behaviours, spatial cognition, temporality

## Abstract

This exploratory study addresses the following question: *Is there an explanatory relationship between the chronological sequence in which individuals explore an environment and the way they subsequently draw a sketch map of that same environment*? To answer it, we conducted a navigation experiment in *La Plaine Saint-Denis* (France) involving 118 participants tracked in real time, and divided into three groups: (1) solo exploration without instruments; (2) solo exploration with a mobile map; (3) collective exploration through a dedicated application. The comparison of the tracking data with the videos of the sketch map making shows that Group 1 participants drew the places they visited in the chronological order of their exploration. This tendency is less significant in Group 2, and absent in Group 3, suggesting that in the absence of a map and/or collective interactions, individuals who draw a sketch map tend to recall the route they have just taken.

## 1 Introduction

In an increasingly digitalized world where navigation depends heavily on technology, understanding how individuals acquire, memorize, and externalize their knowledge of space remains a crucial challenge. This is particularly relevant as navigation tools and practices continue to evolve, potentially affecting how we build and maintain our mental representations of space. While research has extensively documented what elements people include in their maps and how accurately they represent spatial relationships (Kitchin and Blades, 2002), the process through which navigation experience shapes the production of these external representations remains understudied. At the moment, we still do not fully understand how different modes of spatial exploration – from direct environmental experience to technology-mediated navigation and collective exploration – influence the way people externalize their spatial knowledge through the drawing of sketch maps. This gap is significant for both theoretical and practical reasons. While the link between spatial behaviours and cognitive maps is well established (Tolman, 1948; Golledge, 1984), many studies in psychology have primarily focused on the internal structure and accuracy of cognitive maps (e.g., Denis, 2017), often neglecting the embodied and experiential aspects of spatial learning (Waller et al., 2004; Chrastil and Warren, 2012; Ishikawa, 2020). From a practical perspective, as navigation increasingly relies on digital tools, understanding how different navigation conditions influence spatial knowledge externalization becomes crucial for designing more effective navigation aids and spatial learning environments.

In this context, our research specifically examines how different modes of environmental exploration – direct experience, map-aided navigation, and collective exploration – influence the way people externalize their spatial knowledge through sketch maps. By analyzing both the content and the drawing process of sketch maps in relation to participants’ actual navigation patterns, we aim to better understand the relationship between spatial behaviours and spatial knowledge representation.

## 2 Theoretical background

### 2.1 Spatial behaviours and cognitive/mental maps

Spatial behaviour encompasses how individuals interact with and move through their environment, including navigation strategies, route choices, and orientation behaviours. These behaviours both reflect and influence our understanding of space. While early behaviourists of the 20th century subscribed to a simple “stimulus–response” (SR) model, considering only observable actions, the pioneering work of Tolman and Honzik (1930) challenged this view through their experiments on spatial learning in rats. Their findings revealed that animals develop sophisticated mental representations of space rather than merely responding to stimuli – a capacity Tolman (1948) later termed a “cognitive map.” This concept of cognitive mapping proved equally relevant to human spatial cognition. The subsequent research of Lynch (1960) demonstrated how people mentally represent urban environments, identifying five key elements that structure these mental maps: (1) paths; (2) edges; (3) districts; (4) nodes; (5) landmarks. These elements form the basic vocabulary through which people understand and navigate their environment. The existence of cognitive maps has since been validated by numerous studies in neuroscience. Research has identified specialized neurons supporting spatial cognition, including place cells in the hippocampus (O’Keefe and Dostrovsky, 1971), along with grid cells, head-direction cells, and border/boundary cells (Hartley et al., 2014). These findings have also been confirmed in humans (Ekstrom et al., 2003; Jacobs et al., 2013). While these neurobiological findings confirm that our brains maintain spatial representations, as Nadel (2013) notes, these cognitive maps act like, rather than literally look like, geographic maps.

### 2.2 Spatial knowledge

How people acquire and structure their knowledge of space is central to understanding navigation behaviour and spatial representation. Spatial knowledge refers to information about the locations of objects and phenomena, and their relative arrangements; that is, how they are geographically linked to each other. Recent theoretical frameworks have identified several key types of spatial knowledge that develop through environmental interaction. McNamara (2013) identifies four main types: object-place knowledge (memorizing specific locations), route knowledge (memorizing sequences of landmarks and associated actions), environmental shape knowledge (perceiving forms and structures), and survey knowledge (understanding overall environmental layout). The last type is particularly significant as it enables the most sophisticated navigation behaviours, such as creating efficient routes, pointing to non-visible places, and estimating Euclidean distances. While early models proposed that these types of knowledge were acquired sequentially (Siegel and White’s (1975) L-R-S model), subsequent research has revealed a more complex picture. Montello (1998) demonstrated that metric knowledge begins to be acquired from the first exposure to a place, suggesting a more continuous and parallel process. Chrastil and Warren (2014) showed how different types of spatial knowledge – from place recognition to survey understanding – develop concurrently and support each other. Additionally, the means through which people explore space significantly impacts knowledge acquisition. Studies have revealed that while maps aid in understanding locations and distances, they are less effective than direct experience in developing precise route knowledge and orientation skills (Willis et al., 2009), as well as navigation performance and sketch map accuracy (Ishikawa et al., 2008). Additionally, there are a number of factors that impact spatial knowledge acquisition, such as spatial familiarity (Quesnot and Roche, 2015; Quesnot, 2016), gender (Voyer et al., 2007), and culture (Heft, 2013; Quesnot et al., 2024).

### 2.3 Navigation and collective interactions

Navigation involves purposeful movement through space – a process fundamentally different from mere wandering. In this sense, navigation is inseparable from wayfinding and locomotion. As Montello and Sas (2006) rightly point out, the difference between wayfinding and locomotion remains purely conceptual. The embodied nature of navigation has been well-documented. Piaget and Inhelder’s (1948) study highlighted the importance of the body and movement in children’s spatial development, emphasizing how physical interaction with the environment is crucial for forming complex spatial representations. This understanding has been reinforced by recent research showing how bodily experience enhances spatial learning. Ruddle et al. (2011) found that walking, by providing proprioceptive and vestibular information, significantly improves the accuracy of participants’ cognitive maps. Additionally, the research of Chrastil and Warren (2012) indicates that idiothetic information obtained while walking contributes to metric knowledge of the environment, aligning with Waller et al.’s (2004) findings on body-based cues.

However, navigation is rarely a purely individual process. Recent research has revealed the significant social dimensions of spatial learning. As shown by Dorfman et al. (2021), humans develop collective spatial representations of the environment, and the notion of a social cognitive map is increasingly accepted. The research of Dalton et al. (2019) demonstrated that even the mere presence of others influences individual orientation. These social aspects become particularly relevant in the context of technology-mediated navigation. Studies by Reilly et al. (2009), He et al. (2015), and Bae and Montello (2019) have shown how navigation performance is affected by collaboration and group dynamics (see also Curtin and Montello, 2023). More recently, Quesnot and Guelton (2023) demonstrated that collective interactions during environmental exploration notably increased the accuracy of sketch maps produced in groups, compared to those produced by individuals exploring either directly or with a map. This social dimension of navigation is particularly relevant to our study, as it suggests that collective exploration might influence not only how people move through space, but also how they internalize and represent spatial information.

This emerging understanding of navigation as both an embodied and social process raises fundamental questions about how different modes of exploration – whether individual or collective, with or without technological assistance – may influence not only the acquisition of spatial knowledge but also its externalization. While previous research has established the importance of direct experience, navigation tools, and social interactions, we still do not fully understand how these different factors affect the complex process of translating spatial experiences into external representations. This gap is particularly significant given the increasing reliance on digital navigation tools and the growing recognition of navigation as a social activity.

## 3 Objective and research question

Previous research has established how different factors – from navigation aids to social interactions – can influence spatial learning. However, a critical aspect remains understudied: how these factors affect the process through which people externalize their spatial knowledge. The existing literature has primarily focused on the structure, content, and accuracy of cognitive maps, often neglecting the experiential dimension – physical movement in space – which actively contributes to their formation, and thus potentially to their externalization in the form of sketch maps.

While we know that different navigation conditions can affect spatial knowledge acquisition, we do not fully understand how these conditions influence the way people translate their spatial experience into physical representations. This gap is particularly relevant when examining contexts of exploring unknown environments. Given the exploratory nature of this investigation and the lack of previous studies directly examining the relationship between exploration chronology and sketch map creation, we opted for a research question approach rather than formal hypotheses testing, allowing us to investigate potential patterns without preconceived assumptions about the nature of these relationships. *Is there an explanatory relationship between the chronological sequence in which individuals explore an environment and the way they subsequently draw a sketch map of that same environment? If so, what form does this explicative relationship take (linear or non-linear)?*

This question emerges from three key aspects of spatial cognition research: (1) the ongoing debate about how direct environmental experience shapes spatial knowledge acquisition (Sections 2.1 and 2.2); (2) the impact of different navigation aids on spatial knowledge acquisition (Section 2.2); (3) the emerging understanding of navigation as a social process (Section 2.3). To investigate these potential relationships, we designed our study around three distinct navigation conditions:

- Direct environmental exploration without technological assistance;
- Navigation with a mobile mapping application;
- Collective exploration with a shared mapping tool.

This research design allows us to investigate how different modes of environmental interaction might influence both the acquisition and externalization of spatial knowledge.

## 4 Materials and methods

To investigate how different navigation conditions influence spatial knowledge externalization, we designed an experiment comparing three modes of environmental exploration. This study was conducted in a real urban environment, allowing us to examine spatial behaviour and representation under naturalistic conditions. It uses data from a broader experiment previously described in Quesnot and Guelton (2023), which examined collective dynamics in spatial cognition. The present paper, however, addresses a distinct research question and employs different analytical methods.

### 4.1 Study area

**Figure 1:**
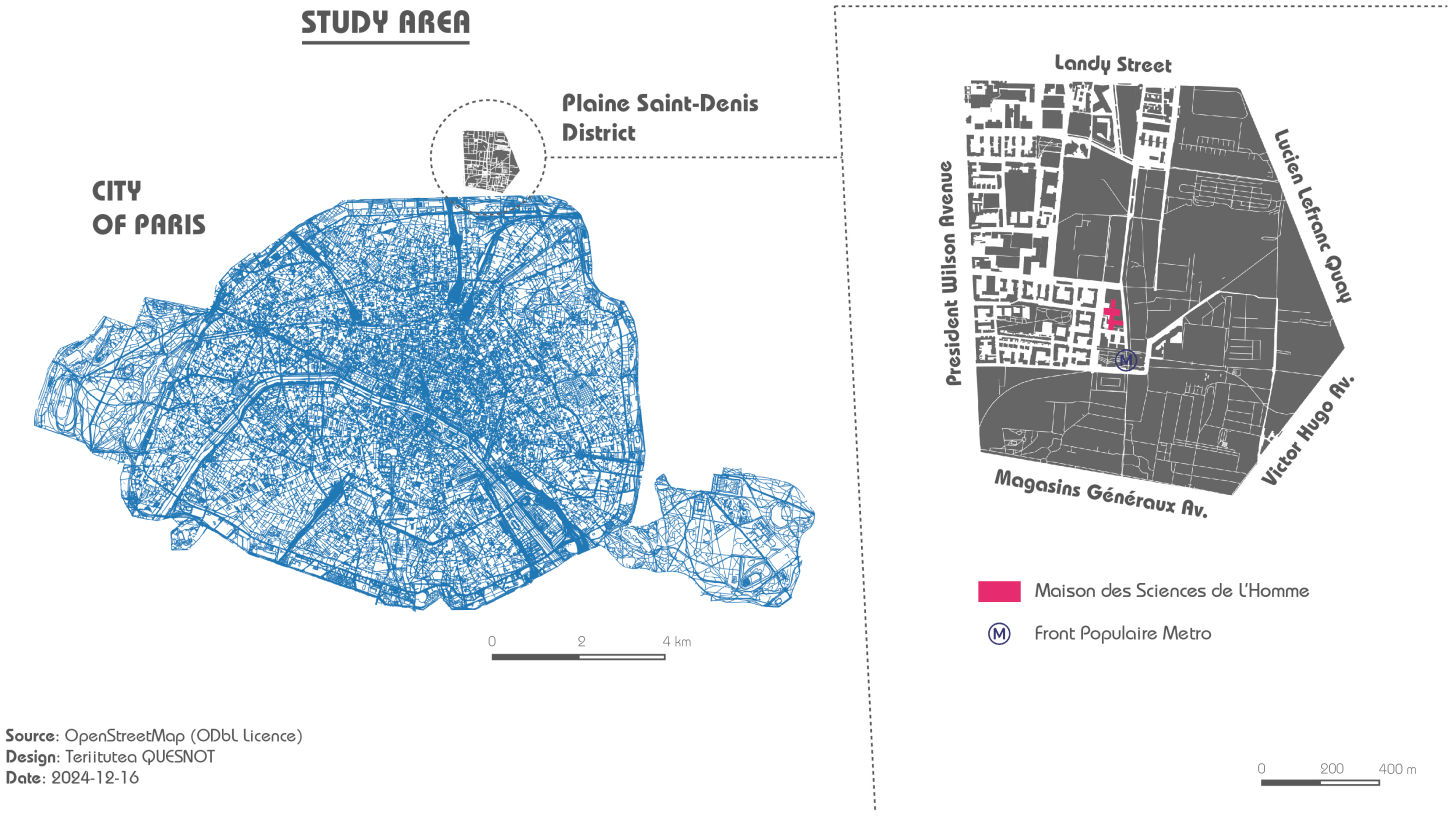
Study area of the experiment.

We sought an urban environment that would allow us to study navigation behaviour under controlled yet realistic conditions. We found a newly renovated urban district in northern Paris (1.5 km^2^) – Plaine Saint-Denis – that matches the following criteria: diversity of places (shops, schools, residences, places of worship, leisure areas, green spaces, etc.); quality of roads for pedestrian navigation (wide and safe); accessibility (proximity to Paris intra-muros); and the fact that the area remains largely unknown. Our study area is bordered by five main roads: *Landy Street* (North), *Lucien Lefranc Quay* (East), *Victor Hugo Avenue* (South-East), *Magasins Généraux Avenue* (South), and *President Wilson Avenue* (West) (Figure 1).

### 4.2 Operationalization of the research question

To examine the relationship between exploration and spatial representation, we developed quantitative measures for both spatial behaviour and sketch map creation. We assumed there is an explanatory relationship between the *time taken to physically reach a place*, and the *time for this same place to appear on the sketch map*. To investigate this, we identified two key indicators: (1) the *duration taken by individuals to reach a place from a designated starting point* during their exploration (independent variable – IV); (2) the *duration they take to draw this same place on their sketch map* following the exploration (dependent variable – DV):

*Duration to physically reach a place from a starting point (IV) → Duration to draw this place on a sketch map (DV).*

These temporal measures allow us to quantify both the exploration process and the externalization of spatial knowledge, enabling us to examine potential relationships between them.

### 4.3 Power analysis

Prior to recruitment, we conducted a power analysis on G*Power to determine the size of our sample. We assumed a classic alpha level of 0.05 and a small effect size of 0.25 considering the exploratory nature of this study. The result delivered by G*Power indicated that a minimum sample size of 34 individuals per group would provide a sufficient power of 80% to detect a small effect size across the study groups.

### 4.4 Sampling and design

Based on this power analysis, we aimed to recruit 40 participants per condition to account for potential data loss. This led to recruiting 118 participants (approximately 40 per group) – 68 women and 50 men – with an average age of 24 years, using university electronic mailing lists. Two conditions were formulated during the hiring process: (1) being at least 18 years old; (2) being unfamiliar with the Plaine Saint-Denis district. Written consents were obtained for each step of the experiment^1^ (i.e., tracking and filming the drawing of the sketch map). Once the experiment completed, each person received a payment of €75 for their involvement.

To examine how different navigation conditions might influence spatial knowledge acquisition and representation, we conducted the experiment under three specific conditions:

- *Condition 1 (direct experiencers)*: participants from Group 1 (21 women and 19 men divided into 8 subgroups of 5 persons) explored the study area *alone* with no devices;
- *Condition 2 (map learners)*: participants from Group 2 (20 women and 20 men divided into 8 subgroups of 5 persons) also explored the study area *alone*, using a dedicated mobile mapping application (mobile map learners);
- *Condition 3 (collective learners)*: in contrast, participants from Group 3 (27 women and 11 men randomly divided into 8 subgroups of up to 5 persons, without regard to gender or spatial skills) explored the study area *collectively* in subgroups using the same application as that of Group 2, enhanced with collective interaction feature.

### 4.5 Devices

#### 4.5.1 Tracking device

To ensure accurate recording of movements, we followed participants’ explorations in real time using a smartphone. The tracking allowed us (1) to ensure that the participants walked within the study area, and (2) to record their movements for later comparison with the drawing of sketch maps. The tracking data was collected using GPS functionality with a sampling rate of one point every 5 s, and stored in GPX format on a secure server.

#### 4.5.2 Navigation device

##### 4.5.2.1 Location map

**Figure 2:**
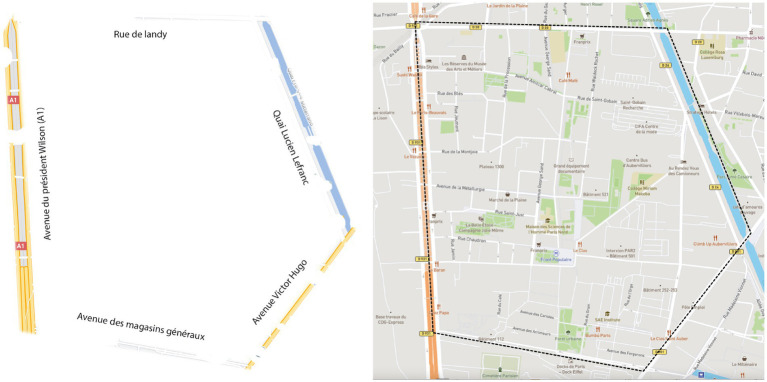
Map given to participants from Group 1 (left) and screenshot of the mapping application used by Group 2 (right).

Before leaving the Paris North Human Sciences Institute (in French, Maison des Sciences de l’Homme – MSH), participants from Group 1 received a paper map that highlights the boundaries of the study area. For the purpose of the experiment, we removed all the geographic elements located within the study area (places, street names, etc.) (Figure 2, Left). This simplified paper map was designed to provide minimal spatial information, allowing us to study navigation behaviour in conditions closest to direct environmental interaction.

##### 4.5.2.2 Mobile mapping application

Participants from Group 2 received a smartphone to find their way while exploring the study area. The mobile mapping application they used displayed a conventional basemap (Figure 2, Right). The application interface provided standard mapping features including zoom capabilities and current location display. The basemap was similar to Google Maps and OpenStreetMap to ensure consistency with commonly used navigation applications.

##### 4.5.2.3 Shared mapping application

**Figure 3:**
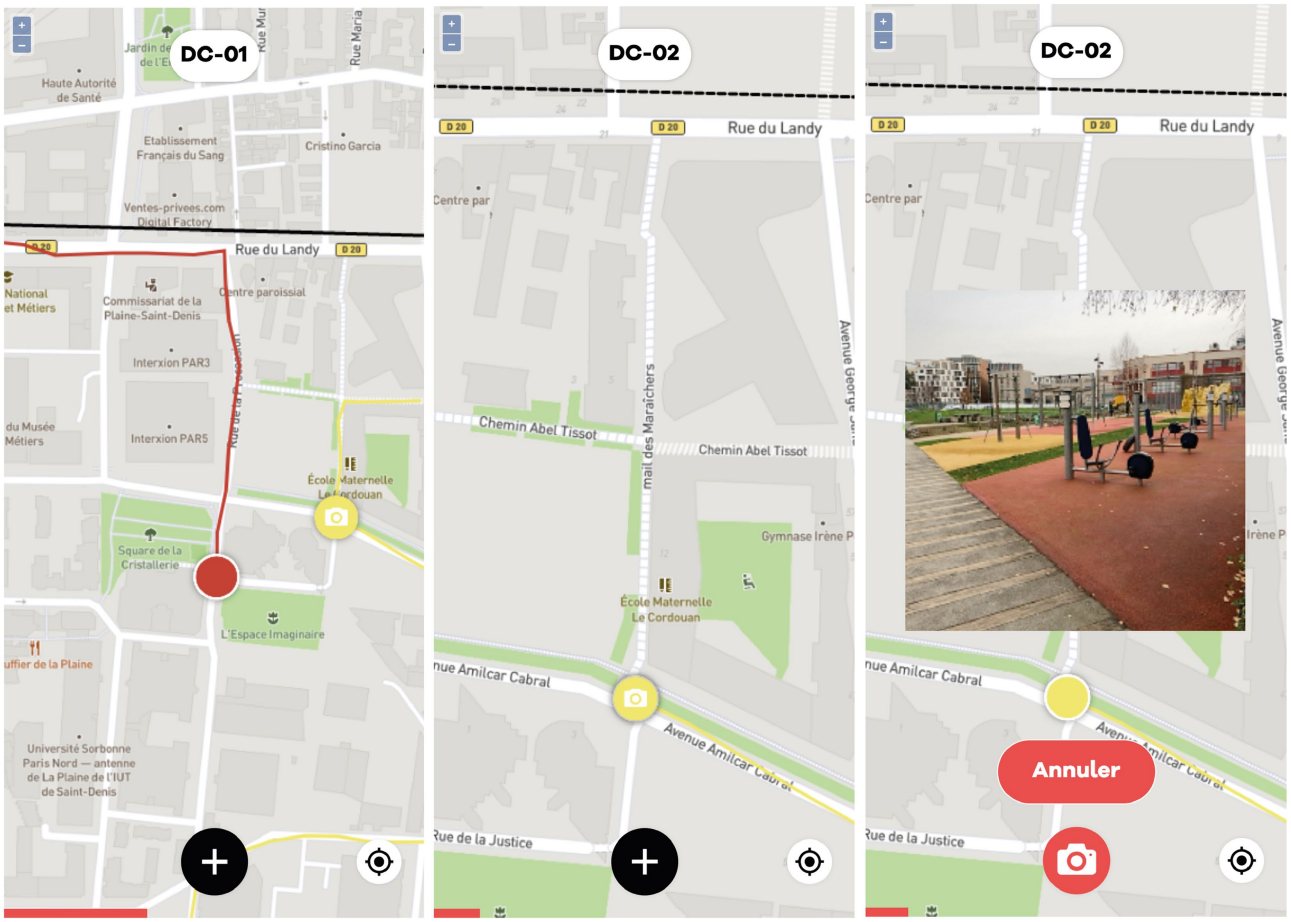
Screenshot of the shared mapping application (Group 3).

Group 3 used the same mobile application as the one used by the previous group, but with collective interaction features. The application was developed by ORBE company’s research team (Paris). Participants were able to visualize in real time the itineraries taken by the other participants of the same subgroup, with no possibility to hide them (refresh rate: 30 s). They could also share geolocated photos which appeared as clickable markers on all subgroup members’ screens (Figure 3). Photo sharing was voluntary and no constraints were given regarding timing or number of uploads. On average, each participant uploaded between 3 and 7 photos during exploration. Although participants were encouraged to coordinate with teammates *via* the app (sharing photos and visualizing routes), no instructions were given on how to organize exploration strategies, divide the area, or assign tasks.

#### 4.5.3 Device for recording the design of the sketch maps

To capture the complete process of sketch map creation, the drawing of the sketch maps after the in-situ exploration was filmed using smartphones that were suspended above the participants to avoid disturbing them. The recording setup consisted of smartphones mounted on adjustable stands positioned approximately 1 meter above the drawing surface, providing a clear overhead view of the entire A4 paper and drawing process.

### 4.6 Procedure

Our experimental protocol was designed to standardize the exploration and sketch map creation process while maintaining ecological validity. The experimental procedure aimed to capture both the spatial behaviour during exploration and the cognitive process of sketch map creation. Each step was carefully planned to minimize potential biases and ensure consistency across all participants.

More specifically, the experiment was conducted between October 2020 and March 2021, and was divided into five main steps:

1) *Reception*: we first received the participants at the MSH. After reviewing the experiment’s objectives with the participants, we secured their written informed consent. Subsequently, we provided them with smartphones to enable real-time tracking.
2) *Instructions*: we gave the following instructions:

“*Somebody you know wants to move into the Plaine Saint-Denis district. She lives too far away to come and have a look herself. She asks you to do some exploring and draw a map for her. Please indicate any useful landmarks when you are out exploring the district so that she can see what the environment is like.*” [Translated by the authors].

The instruction for the third group included a supplement:

“*To meet these objectives, you will explore the area with 4 other participants – but with everybody at a distance from one another – by using the interactive smartphone application that has been given to you. The map on the mobile phone enables you to share your routes and your photos to interact with your teammates. Observe the district, find, and photograph places of interest in coordination with your teammates.*” [Translated by the authors]

3) *Equipment*: after receiving instructions, participants were outfitted with smartphones enabled for tracking purposes. Those in Group 1 were given a paper location map, whereas participants in Groups 2 and 3 were given demonstrations and instructions on using the mobile mapping application. Each participant was allowed time to familiarize themselves with their respective navigation tools.
4) *Exploration*: participants navigated the Plaine Saint-Denis district on foot for approximately 1 hour before returning to the MSH. The one-hour duration was chosen based on pilot studies showing it provided sufficient time to explore the main areas while maintaining participant engagement. Those in Group 3 conducted their exploration in small and randomly assigned subgroups, consisting of three to five individuals each. During exploration, experimenters monitored participants’ locations remotely through the tracking system to ensure safety and compliance with study boundaries.
5) *Sketch maps*: upon returning to the MSH, participants were individually filmed while creating their own sketch maps on an A4 paper. They were given the liberty to choose and change markers as needed. Participants from Group 3 drew their individual sketch map independently, without discussion or input from subgroup members. To minimize potential influence, a standardized setup was used: the experimenter started the video recording and then exited the room to avoid any interference with the participants’ activities and eliminate any potential bias in the drawing process. The video recording of the sketch map creation process was crucial for this study, as it allowed to analyse the temporal aspects of how participants externalized their spatial knowledge.

All procedures were conducted in accordance with the ethical standards of the authors’ institutions. While formal ethical review was not required under French regulations for this type of non-invasive behavioural study, all participants provided informed consent, and their data were anonymized to protect their privacy.

### 4.7 Data collection and analysis methods

Our analysis methodology was designed to capture both the spatial and temporal aspects of participants’ exploration and sketch map creation. We employed a multi-faceted approach, combining qualitative analysis of sketch maps with quantitative analysis of tracking data and video recordings.

#### 4.7.1 Sketch maps analysis

Our analysis focuses primarily on the chronological order in which landmarks appear during the sketch map creation process, rather than on the spatial accuracy of these representations. This approach allows us to investigate the relationship between physical exploration patterns and spatial knowledge externalization, while minimizing the potential confounding effects of individual differences in drawing ability. By concentrating on when and in what order participants draw elements, rather than how accurately they represent them, we can better isolate the cognitive aspects of spatial memory externalization from the variability introduced by graphomotor skills.

First, the sketch maps were initially analysed manually to identify the primary elements depicted in the drawings. To facilitate the analysis while adhering to our research question, only places represented as toponyms and/or explicit symbols (e.g., a cross for a church or an -M- for a subway station) were included. Transitory features (e.g., construction sites, parked cars, etc.) were excluded from the outset.

To enable systematic analysis of spatial patterns, a global geographic database in shapfile format (SHP) was then created concurrently with the content analysis of the drawings using the Geographic Information System QGIS, version 3.16. Only explicitly geolocatable places (i.e., those that can be associated with precise geographic coordinates) were included in this database. After that, an individual SHP database was created for each participant. We used those databases in conjunction with tracking data to estimate the time it took for an individual to reach each visited place from the MSH (IV – independent variable).

Finally, to ensure reliable temporal measurements of sketch map creation, video analysis was conducted using QuickTime, which allowed for precise timing of landmark appearances on sketch maps. Each video was analysed independently by two researchers to ensure reliability, with any discrepancies resolved through discussion. Specifically, we measured DV (dependent variable) – the duration required for a place to appear on a sketch map – by reviewing the recorded drawing sessions sequentially, and noting the emergence time of each previously identified landmark. To standardize our temporal measurements, we marked the beginning of an entity’s construction at the first appearance of either its toponym’s initial letter or the first line of its geometry. In this analysis, we standardized all landmark occurrence times to “Time 0” as a reference point, instead of timing them relative to the appearance of preceding landmarks.

#### 4.7.2 Tracking data analysis

In order to ensure the quality and reliability of our movement data, we implemented a rigorous validation process. Tracking data were screened for anomalies such as unrealistic speeds or positions. Any identified anomalies were cross-checked with video recordings and participant reports to determine their validity.

As a matter of fact, our tracking protocol generated rich spatiotemporal data. Participant location data were collected using the tracking function of their smartphones, which was saved in GPX format and stored on a dedicated server. These data were subsequently imported into QGIS 3.16, and analysed in the form of points (track points) and lines (tracks). The track points contained time stamps, which enabled the calculation of travel times and walking speeds for each participant. Meanwhile, the linear tracks provided data on the distances covered and the overall route coverage rate for each main group. To quantify coverage, the study area was partitioned into a grid with cells measuring 50 by 50 metres, a spatial resolution chosen based on the average time taken by participants to move across such a cell (around 1 min). Following this, the tracks were segmented to calculate the average number of route sections per grid cell for each main group.

In addition, to quantify participants’ progression through the environment, we used the individual SHP databases and corresponding tracking data, alongside the temporal controller of QGIS 3.16, to measure the time (in seconds) it took for participants to physically reach the places they had drawn on their sketch maps, from the MSH. This measurement process involved: (1) Identifying when a participant first came within 20 meters of each landmark they later drew; (2) Calculating the elapsed time from departure at MSH to this first encounter; (3) Cross-validating these timestamps with the GPS tracking data.

### 4.8 Statistical considerations

Given the complex nature of our data, we carefully evaluated the most appropriate statistical approach. In the preliminary analysis (Section 5.3.1), scatter plots indicated a non-linear relationship between IV and DV across all groups. To better capture this non-linearity, we used a Generalized Additive Mixed Model (GAMM) with spline terms (thin plate). This approach was chosen for its ability to model complex, non-linear relationships, while accounting for the hierarchical structure of our data (multiple observations per participant). We reviewed the conditions for applying this model as outlined in Section 5.3.1, which involved assessing linearity through scatter plots, and accommodating non-linear trends with splines in the GAMM. The random effects structure of the GAMM accounted for intra-subject variability due to the repeated measures design, where each subject provided multiple observations. While many statistical models require the assumption of normality of residuals, this criterion is actually not essential for GAMMs employing spline terms. Such feature is beneficial for dealing with skewed or heteroscedastic data, often encountered in real-world scenarios. It is also suitable for exploratory studies like ours, where the primary aim is to investigate potential relationships and trends, rather than to confirm specific hypotheses with precision. The GAMM analysis was performed using R software (version 4.2.3) with the “mgcv” package (version 1.9.0). Model diagnostics, including residual plots and checks for multicollinearity, were conducted to ensure the validity of our results.

## 5 Results and analysis

Our analysis focuses on three main aspects: the characteristics of the sketch maps produced, the spatial behaviours observed during exploration, and the relationship between these two elements. We will present our findings in this order, culminating in a statistical analysis that explores the potential links between exploration patterns and sketch map creation. However, before delving into the results, it is important to note some challenges encountered during the experiment. We faced some difficulties, including bad weather (snow and rain), disconnection of the tracking servers, misunderstood instructions, last-minute cancellations, and video recording problems. Key moments of the experiment were therefore impacted: (1) allocation of smartphones and understanding of the instructions; (2) in-situ explorations; (3) video-recording of the sketch maps. These difficulties resulted in a disparity in the number of participants between the three groups. Additionally, some tracking data, sketch maps, and videos proved to be unusable (absence of places, illegible toponyms, heads hiding the sketch maps during the drawing phase, missing path sections, etc.), bringing the analysable data to 101 individuals – 33 from Group 1, and 34 each in Groups 2 and 3. Despite this loss (15%), the study remains consistent with the parameters of the predetermined statistical power analysis detailed in Section 4.3.

### 5.1 Sketch maps

#### 5.1.1 Spatial dimension

**Figure 4:**
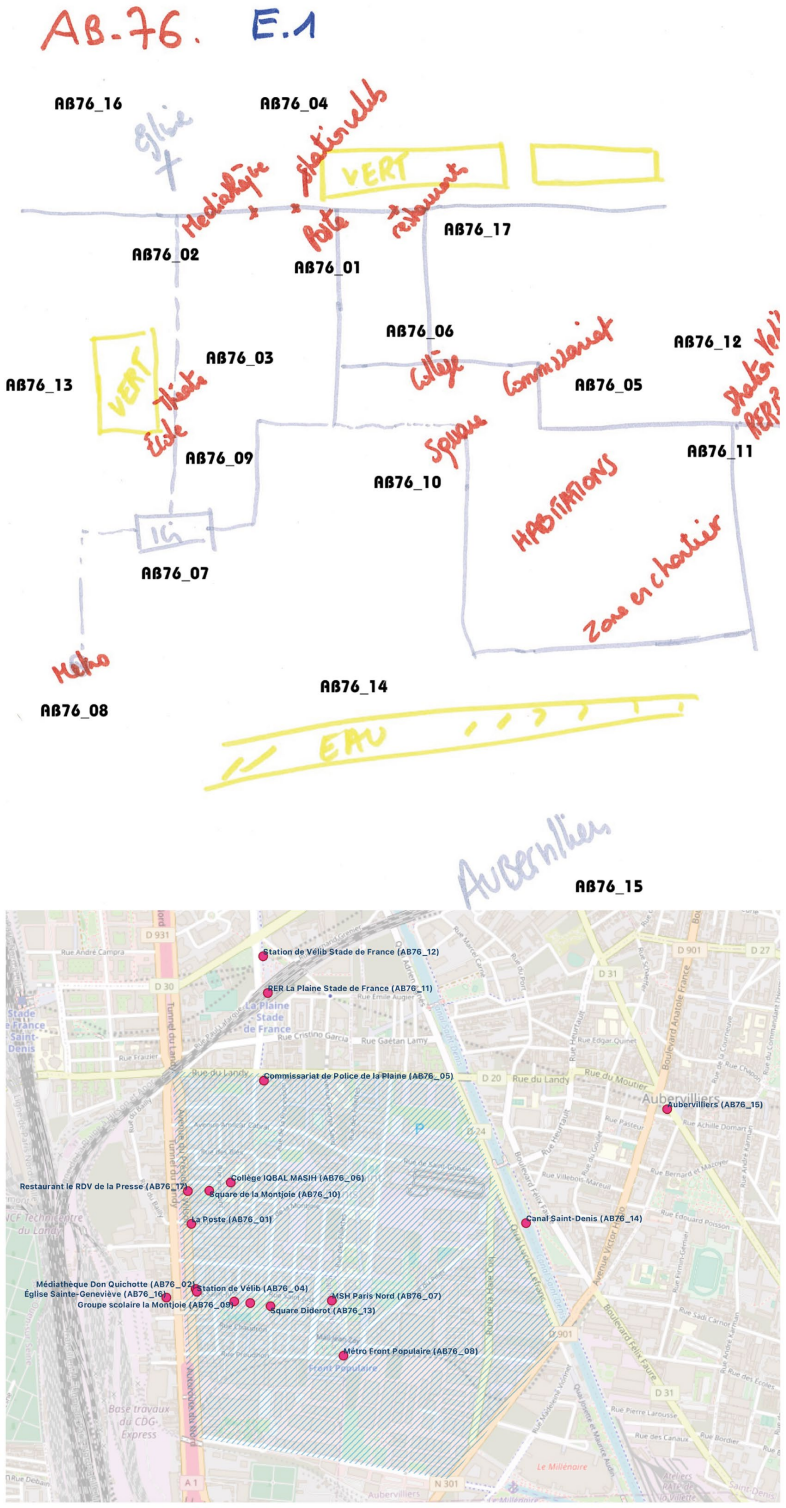
Tagged sketch map (up) and related geographic data of participant A76 displayed on QGIS (down).

Our analysis of sketch maps followed a systematic three-stage process. We first identified all recognizable elements, and then verified the matches between them and their physical counterparts, to allocate a unique alphanumeric identifier (Figure 4, Up). The final step was the creation and the enrichment of a landmark database that contains all the visited places. This global database was then split into individual databases specific to each participant (Figure 4, Down).

To illustrate our analytical approach, we will refer to the case study of participant AB76 from Group 1. This participant’s sketch map outlines 17 distinct entities (Figure 4, Up). Among these, some are clearly labelled, like the Metro (reference AB76_08) and the police station (reference AB76_05). Others are represented through more implicit references. For example, the term “Ici” (“Here” in English, reference AB76_07) is used to denote the MSH. Additionally, the placement of some entities was inferred contextually. For instance, the word “Vert” (“Green” in English, reference AB76_13) is actually an indicative label for “Square Diderot,” which is located next to the “La Belle Étoile” theatre. It is also noteworthy that several entities in the map could not be precisely identified or classified due to their ambiguous or generic nature, such as the term “Habitations” (residences in English).

Applying this analytical framework, we initially identified 1,166 entities: 387 attributed to Group 1, 446 to Group 2, and 333 to Group 3 (Table 1). However, these totals were refined post-evaluation against the tracking data. Places that were located beyond the participant’s route were systematically omitted from the conclusive dataset. “Aubervilliers” (“AB76_15”) serves as a relevant example: while it was referenced by participant AB76 as a global landmark (Figure 4, Up), its status as an outlying city rendered it external to the focused analysis.

**Table 1:** Number of entities identified on sketch maps.

Group	Prior the tracking analysis (unrefined)					After the tracking analysis (refined)				
	Total number of entities	Average number of entities	SD	Min	Max	Total number of entities	Average number of entities	SD	Min	Max
1	387	11.72	5.06	3	26	329	9.96	4.74	3	24
2	446	13.11	6.45	4	27	395	11.61	6.25	3	25
3	333	9.79	4.75	3	22	283	8.32	4.60	2	19
Total	1,166					1,007				
Mean	388.66	11.54		3.33	25	335.66	9.96		2.66	22.66

**Figure 5:**
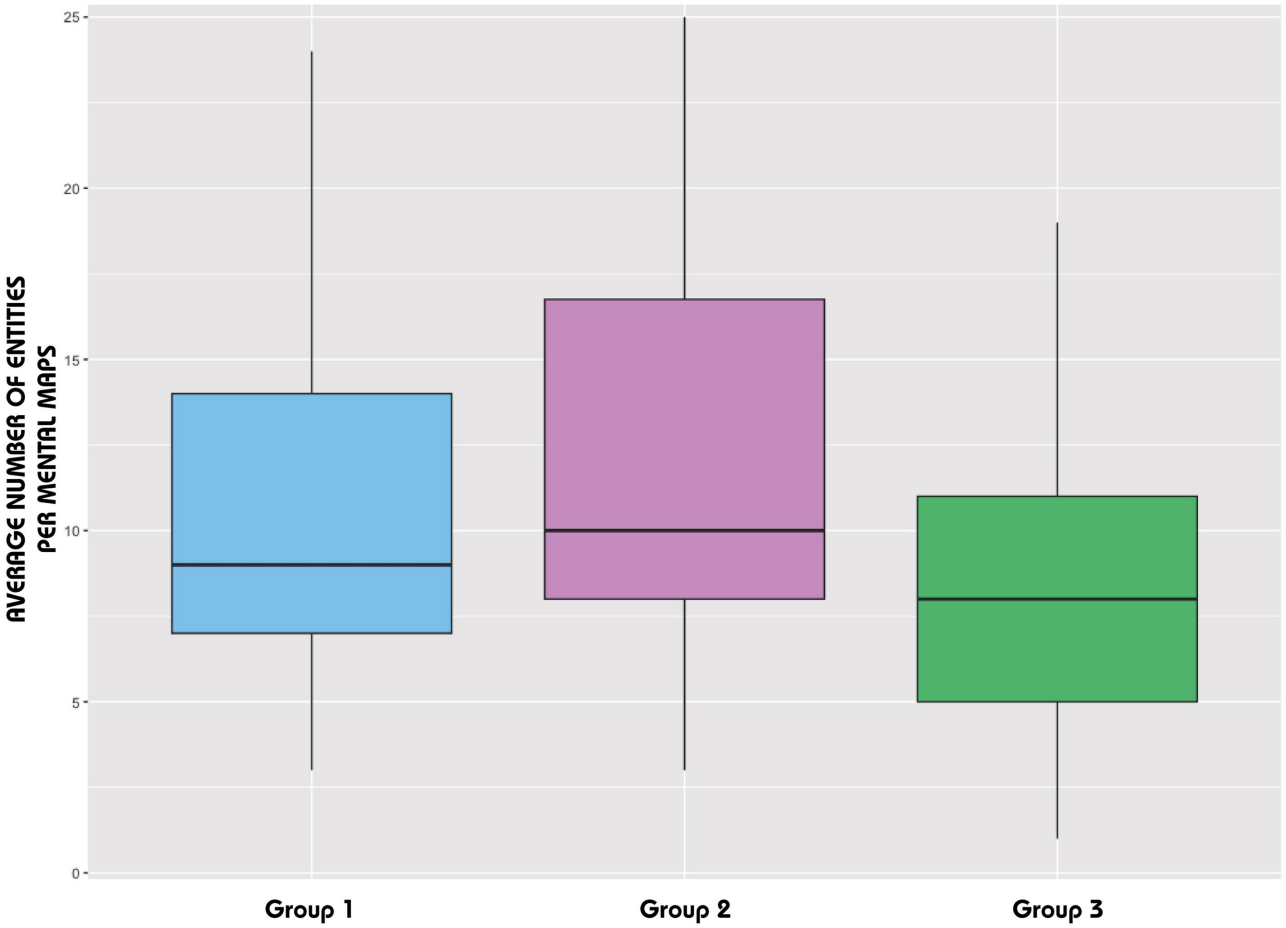
Distribution of the average number of entities per sketch map (after the tracking analysis).

Statistical analysis of these refined data revealed several patterns. The corresponding standard deviations – 4.74 (Group 1), 6.25 (Group 2), and 4.60 (Group 3) – show greater variability within Group 2. Since the distribution in this group does not follow a normal distribution (Shapiro–Wilk test *p-value* = 0.03), we relied on the analysis of the interquartile ranges, which suggests that there is no significant difference between the three groups in terms of content (Figure 5).

#### 5.1.2 Temporal dimension

**Figure 6:**
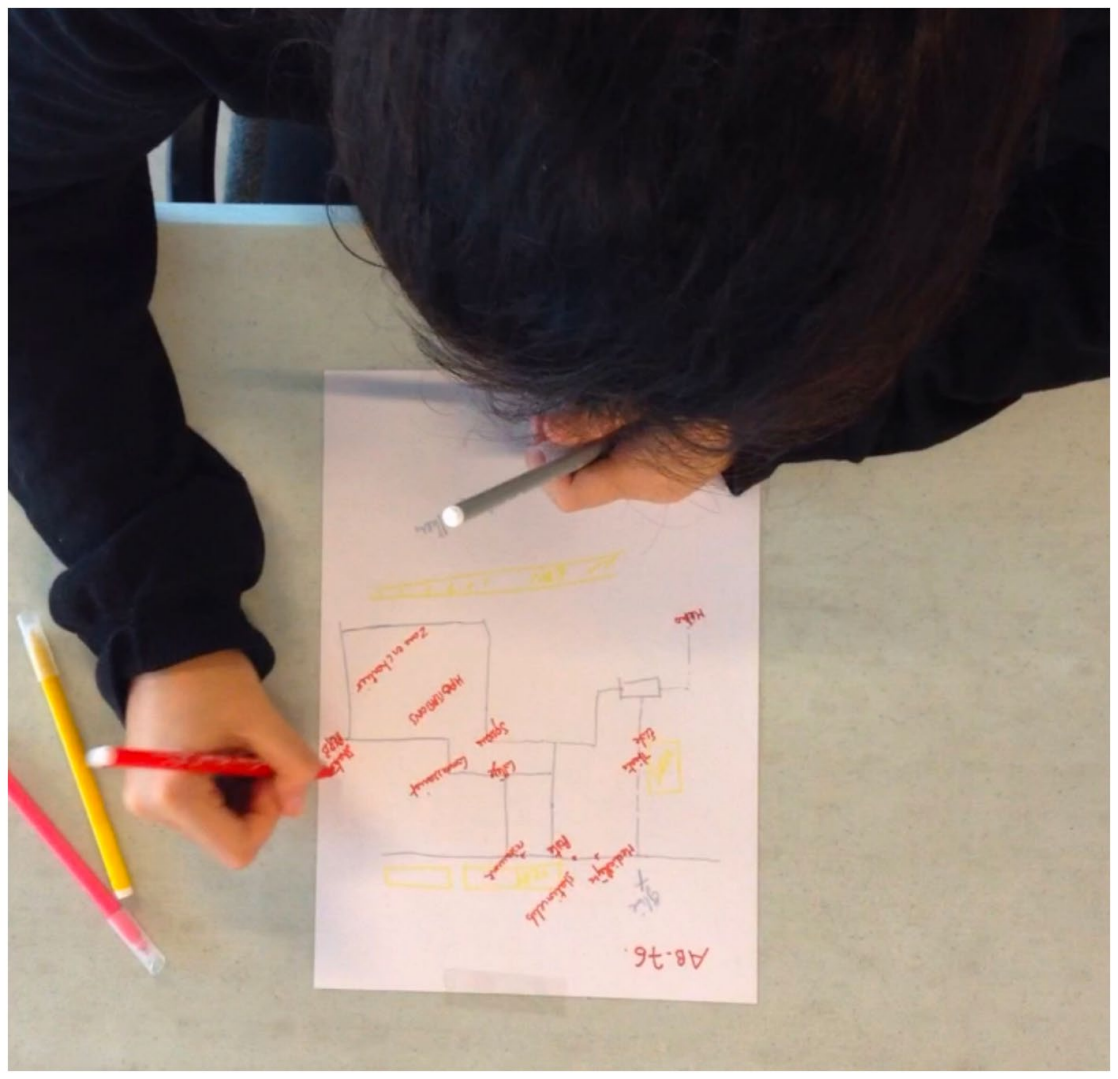
Excerpt from the video of participant AB76.

To understand how participants constructed their sketch maps over time, we recorded the time of first occurrence for each entity previously listed (Figure 6). Using standardized coding procedures, we marked the beginning of the entity’s construction, whether it was the toponym’s initial letter or the first line of its geometry. This task required several reviews to achieve precise annotation. In some cases, full video annotation was prevented by participants obscuring the camera’s view while drawing. These instances led to the exclusion from the video dataset, despite the usability of the corresponding sketch maps.

Analysis of completion times revealed distinct patterns across groups. The mean completion times, as derived from our analysis, are in agreement with the average number of entities identified on the sketch maps (Table 2). Group 2, who averaged 13 entities per drawing, also took the longest time to complete their sketch maps, averaging 12 min and 30 s each. In contrast, Group 3 finished the maps more quickly, averaging 9 min per drawing, but included fewer entities, with an average of 10 per map. Group 1 displayed metrics that fell between the two extremes.

**Table 2:** Average time for completing sketch maps

Group	Average time for completing the sketch maps (seconds)	SD	Min	Max
1	638.63	329.06	150	1,680
2	749.55	340.98	255	1,695
3	532.94	210.20	180	840

Please, note that this is the time required to display the last entity on the map. Subsequent events (typically, putting pens aside when the sketch map is completed) are excluded from the time calculation.

**Figure 7:**
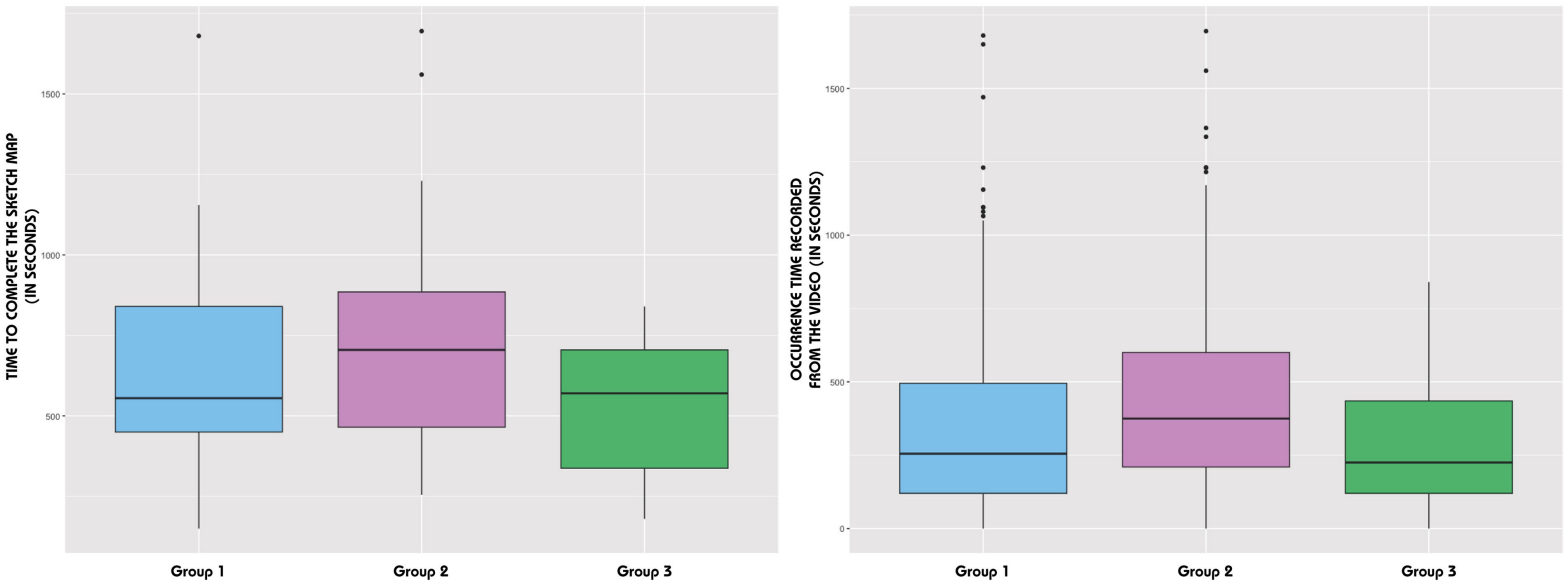
Distribution of the time to complete the sketch map (left) and distribution of the occurrence time recorded from the videos (right).

Standard deviations indicate reduced variability within Group 3 (Table 2). The distribution for this group deviates from a normal distribution, as evidenced by a Shapiro–Wilk test *p-value* of 0.03, which contrasts with the normal distributions observed in the other two groups. Interquartile range analysis reinforces this finding, revealing no significant disparities in completion times across all groups (Figure 7, Left). This pattern of non-significant variation holds when considering the occurrence times where the interquartile comparisons across the three groups also demonstrate a lack of significant difference (Figure 7, Right).

#### 5.1.3 Duration to draw a place on the sketch map (DV – dependent variable)

**Figure 8:**
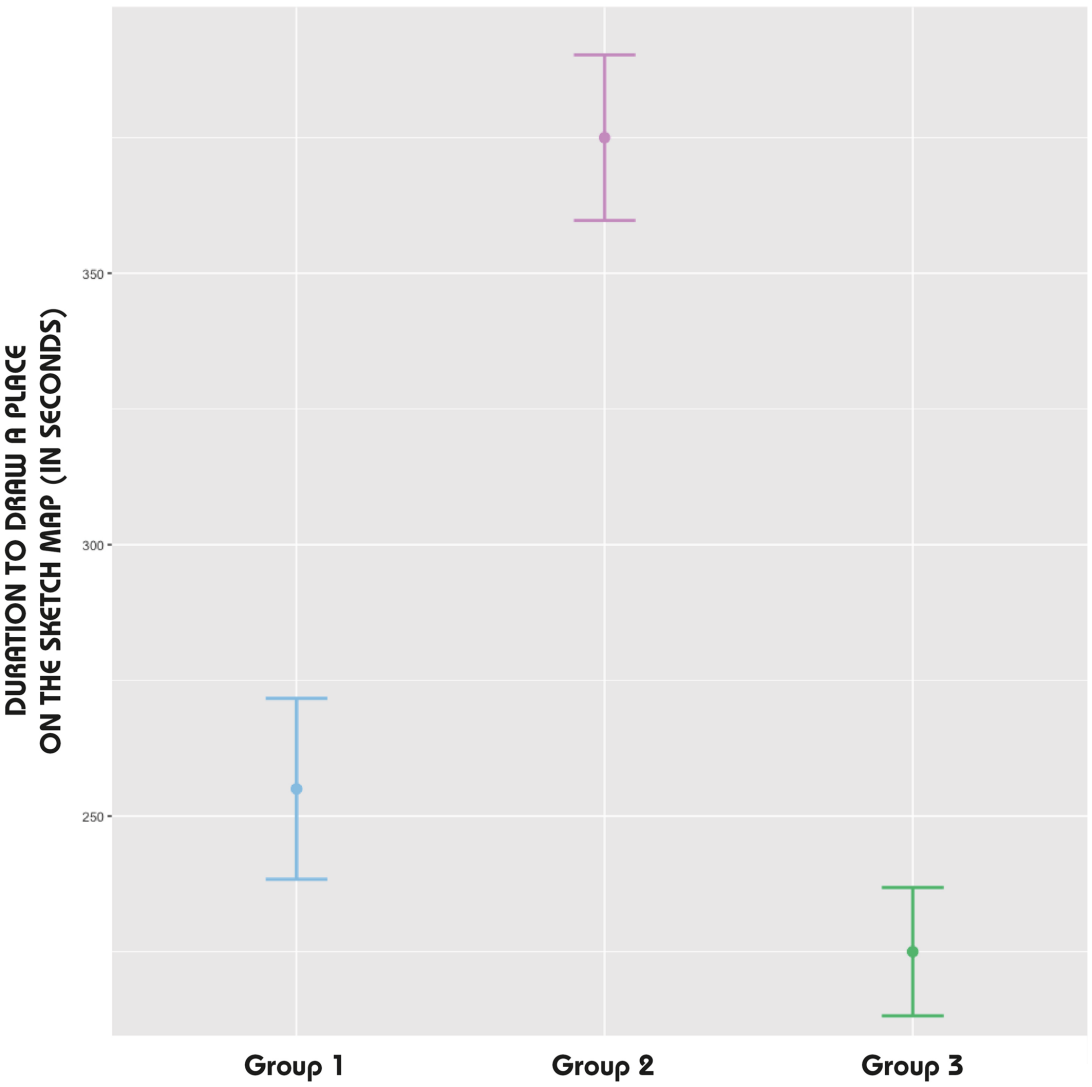
Error bars: duration to draw a place on the sketch map (DV).

While accuracy is an important aspect of sketch maps, our focus on duration allows us to explore the temporal relationship between exploration and spatial knowledge externalization, which is central to our research question. Analysis of drawing durations revealed distinct patterns across groups. The analysis of the duration taken by participants to draw places (DV) was guided by the results of the Shapiro–Wilk test, which indicated a non-normal distribution of data for all groups (*p-value* < 0.05). Consequently, the median was used as a central tendency measure rather than the mean (Figure 8). In examining the patterns across the groups, it was found that Group 1 had a median drawing time of 255 s with a *Standard Error* (SE) of 16.67. Group 2 showed a longer median duration of 375 s (*SE* = 15.26) compared to the other groups. In contrast, Group 3 had a shorter median duration of 225 s (*SE* = 11.82).

### 5.2 Spatial behaviours

#### 5.2.1 Descriptive statistics

**Figure 9:**
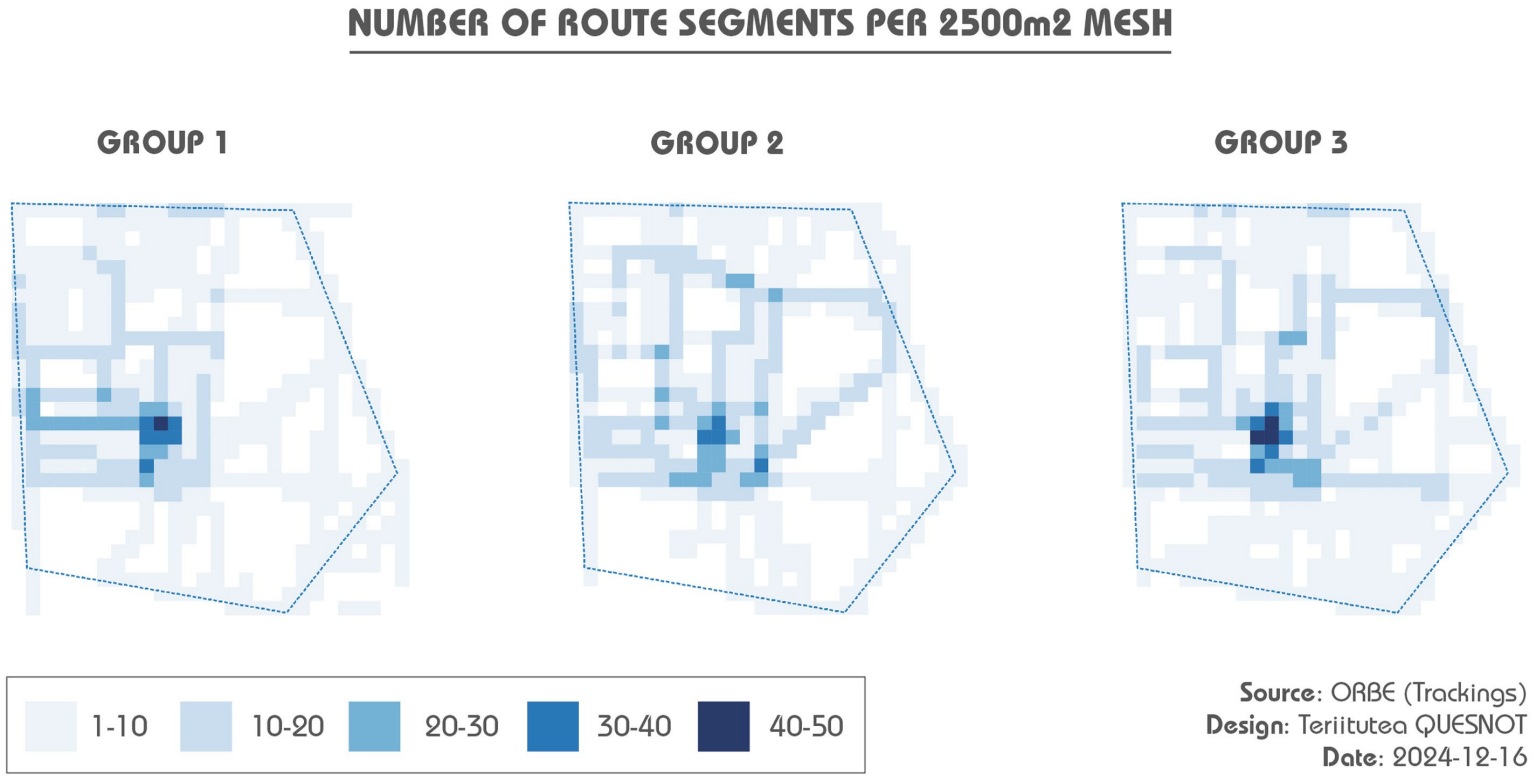
Density maps of completed routes. The itineraries taken outside the study area have not been mapped.

Analysis of the tracking data revealed distinct patterns of environmental exploration across groups. It was used to characterize the spatial behaviours, details of which are included in Table 3. On average, Group 1 dedicated more time to exploration and covered longer distances than the others. Group 3 participants, however, favoured shorter routes in comparison with their counterparts from Groups 1 and 2. Remarkably, participants navigating without assistance strayed outside the study area. Despite the anticipated reliance on the mobile mapping app, two participants from Group 2 unexpectedly covered substantial distances outside the district, reaching 1,180 m and 1,600 m away. Only participants from Group 3 remained within the study area. Furthermore, as we can see from the density maps (Figure 9), Group 3 had a more extensive average area coverage than the other groups, and all participants avoided the southern sector of the district demarcated by Proudhon and Gardinoux Streets. The highest activity density for all groups was predictably located near the MSH, the experiment’s starting and ending points. All these descriptive statistics provide a foundation for understanding the differences in exploration patterns between groups, which may influence the subsequent sketch map creation process.

**Table 3:** Descriptive statistics of the individual (Groups 1 and 2) and collective (Group 3) explorations.

Group	Route time (mins)				Distance (meters)				Average speed (km/h)	Number of routes outside the area	Average distance outside the area (meters)	Area covered (50 m x 50 m mesh)
	Average	SD	Min	Max	Average	SD	Min	Max				
1	71	15	15	95	4958.30	1,080	1,549	6,678	4.20	13	1,683	473
2	64	14	21	72	4628.22	590	2,984	5,930	4.30	2	1,390	454
3	64	14	20	70	4447.04	875	2,378	6,021	4.10	0	0	503
Average	66.33		19	79	4677.85		2,304	6,210	4.20	5	1024.33	476.66

#### 5.2.2 Duration to reach a place from the MSH (IV – independent variable)

**Figure 10:**
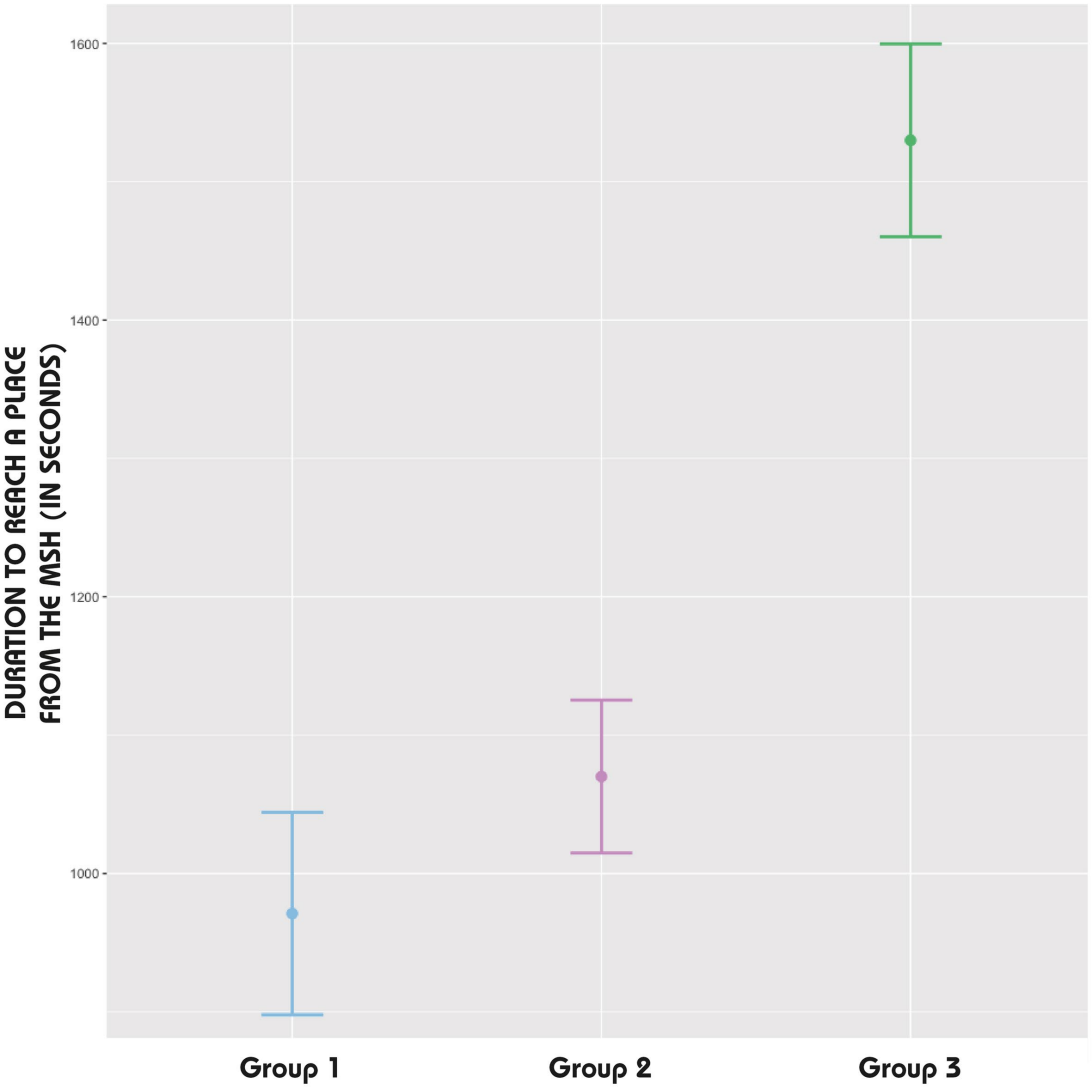
Error bars: duration to reach a place from the MSH (IV).

The duration taken by participants to reach places from the MSH (IV) was influenced by the non-normal distribution of the data, as indicated by the Shapiro–Wilk test (*p-value* < 0.05 for all groups). Therefore, the median was also used as a more appropriate measure of central tendency than the mean (Figure 10). Specifically, Group 1 had a median duration of 971 s (*SE* = 73.17) to physically reach a place, which was faster than Group 3, with a median duration of 1,530 s (*SE* = 69.74). In contrast, Group 2 exhibited a lower median duration of 1,070 s (*SE* = 55.26).

### 5.3 Confronting spatial behaviours and sketch maps

#### 5.3.1 GAMM: preliminary checks

**Figure 11:**
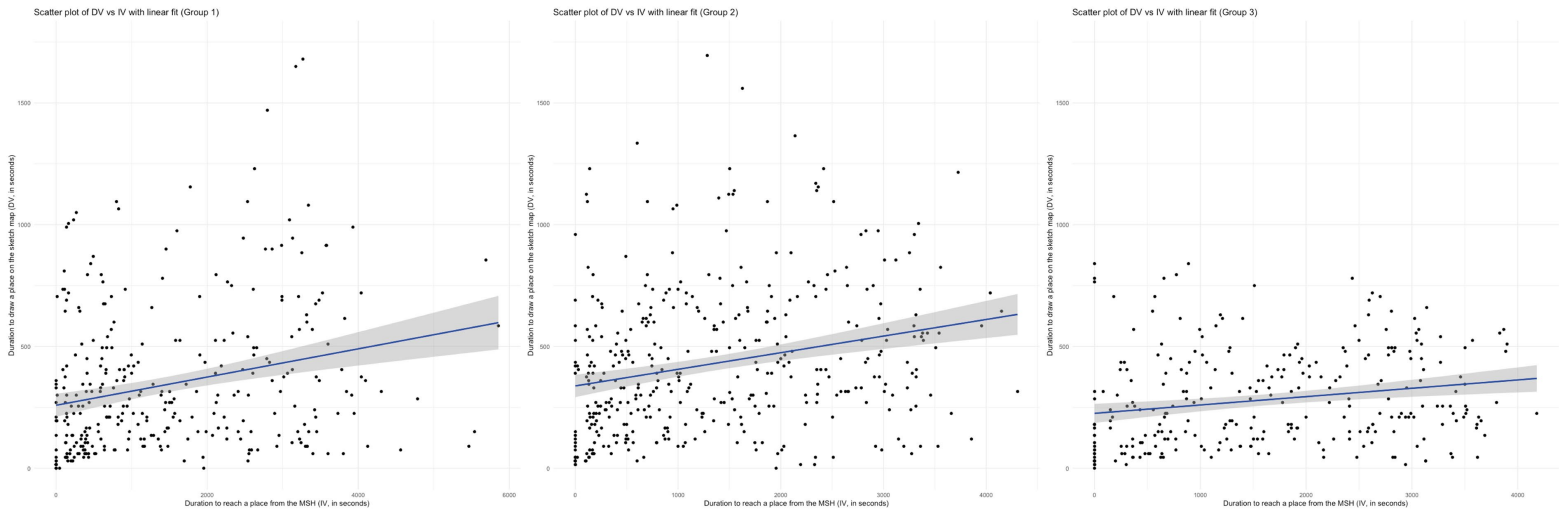
Scatter plots of DV vs. IV with linear fit for Groups 1 (left), 2 (middle) and 3 (right).

Before fitting the GAMM, we examined the assumptions necessary for a linear modelling. We used scatter plots to assess the linearity of the relationship between IV and DV. As Figure 11 - Left shows, Group 1’s scatter plot with a linear fit does not really align with the data. The linear model underestimate the DV at higher values of IV. This non-linear pattern suggested potential non-linear relationship and/or the presence of other contributing variables that are not captured by IV alone. In contrast, Group 2 displayed a more consistent pattern with the linear fit, albeit still with considerable scatter. This indicates once again that non-linear elements and/or other variables affect DV (Figure 11, Middle). Lastly, Group 3 presents a scatter plot where the linear fit appears to be a better match for the lower range of IV, but diverged as IV increased (Figure 11, Right). The data points for higher IV values show greater variability around the linear fit, which implies that the relationship between IV and DV may not be fully explained by a simple linear model for this group. In other words, the relationship between the duration to reach a place from the MSH, and the duration to draw it on a sketch map, is likely to be more complex, and requires a non-linear modelling approach.

#### 5.3.2 Model fitting and evaluation

To better capture the complexity of the relationship between exploration and sketch map creation, we fitted Generalized Additive Mixed Models (GAMMs) using spline terms to allow for non-linear trends, while accounting for individual-level variability.

**Figure 12:**
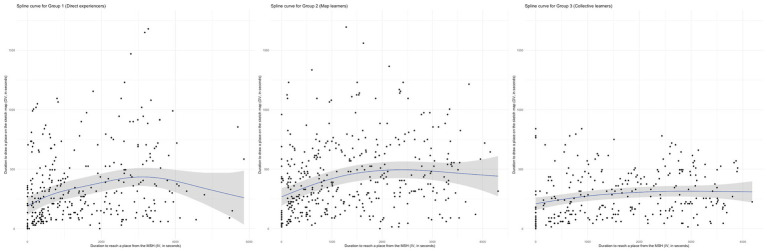
Splin curves with confidence intervals (in grey) for Groups 1 (left), 2 (middle) and 3 (right).

Table 4 summarizes the key statistics for each model. For Group 1, the intercept is 318.60 s (SE = 32.84), and the spline shows an effective degrees of freedom (edf) of 3.179, indicating a complex non-linear relationship between the time to reach a place (IV) and the time to draw it (DV). The model is statistically significant (*F* = 15.91, *p* < 2e-16) and explains 7.19% of the variance (adjusted *R*^2^ = 0.0719). This relationship is visually represented in Figure 12 - Left, where the spline curve shows a clear rise and fall over the time axis, contrasting with the linear fit presented earlier in Figure 11, Left.

**Table 4:** GAMM model summary statistics for the three groups.

Group	Intercept	SE intercept	edf	*F*-value	*p*-value	Adjusted *R*^2^	Scale estimate
1	318.60	32.84	3.179	15.91	< 2e-16	0.0719	41,479
2	405.57	29.52	2.502	16.46	< 2e-16	0.0833	58,681
3	279.62	18.79	1.844	5.036	0.00494	0.0492	29,809

Group 2 displays a slightly less complex curve (edf = 2.502), but with similarly strong significance (*F* = 16.46, *p* < 2e-16). The intercept is higher at 405.57 s (SE = 29.52), and the adjusted *R*^2^ value is slightly improved at 0.0833. The corresponding curve in Figure 12, Middle confirms a non-linear relationship, though the transition is smoother than that of Group 1.

In contrast, Group 3 shows a simpler non-linear pattern (edf = 1.844), with a lower intercept (279.62 s, SE = 18.79), a significant but less pronounced fit (*F* = 5.036, *p* = 0.00494), and the smallest amount of explained variance (adjusted *R*^2^ = 0.0492). This modest relationship is echoed in the near-flat curve shown in Figure 12 - Right, and the divergence from linearity in Figure 11, Right is notably less distinct than in the other groups.

Despite the modest adjusted *R*-squared values, our results maintain statistical validity, with a power of 80% to detect even weak signals. This aligns with our exploratory aim of discovering potential patterns and generate hypotheses in a context where the complex nature of the phenomena does not always allow us to get a high-explanatory power.

#### 5.3.3 Interpretation

The GAMM analysis revealed distinct patterns in how different navigation conditions influenced spatial knowledge externalization. It shows a non-linear relationship between the duration individuals take to physically reach a place (IV), and the time they spend to draw that place on their sketch map (DV).

For direct experiencers (Group 1) and map learners (Group 2), we observed a time-dependent pattern: the longer it takes someone to reach a given place from the MSH, the longer this place takes to appear on the corresponding sketch map. However, after a certain level, the increase in navigation time no longer corresponds to a greater drawing time. In contrast, collective learners (Group 3) demonstrate a more consistent pattern, where the drawing time does not vary as much with the navigation time.

These differences suggest that navigation conditions fundamentally affect how spatial knowledge is processed and externalized. While the GAMM analysis reveals significant non-linear relationships between exploration time and sketch map creation time across all groups, it is important to note that these models explain only a portion of the observed variability. This suggests that other factors, beyond the scope of our current analysis, may also play important roles in the process of translating spatial experiences into sketch maps.

## 6 Discussion

Our study highlights how different modes of environmental exploration influence the way people externalize their spatial knowledge through sketch maps. It reveals varying degrees of non-linearity in the relationship between exploration time and sketch map creation across the three experimental groups. The direct experiencers (Group 1) showed the strongest temporal correlation, while the map learners (Group 2) demonstrated a weaker relationship, and the collective learners (Group 3) showed almost no temporal correlation. These findings provide evidence for an explanatory relationship between the chronological sequence of exploration and sketch map creation, but most importantly, this relationship varies depending on the navigation conditions.

The GAMM analysis shows distinct cognitive processes across navigation conditions. It demonstrates a varying extent of non-linearity (expressed as *edf*) across the three groups. The model fitting for Group 1 illustrates a bell-shaped curve peaking around 50 min of exploration, then symmetrically decreasing towards approximately 95 min for the slower walkers. The rising phase suggests that individuals in the first group depicted places they visited in chronological order of their walk up to about 50 min. Notably, the average exploration time for Group 1 is 71 min, indicating an average decline of about 20 min during which the trend reverses: the places drawn on the sketch maps of Group 1 tend to be those visited earlier in the exploration. Assuming that individuals in Group 1 recalled their route while making their sketch map, this second phase logically stems from the exploration’s start and end point being the same. In this context, the MSH appears to be the primary anchor point (Golledge, 1984; Couclelis et al., 1987), guiding both navigation and spatial knowledge acquisition: participants in Group 1 seemingly relied on this landmark to chronologically recall the route they took – and thereby the places they visited – during the exploration. The strong temporal correlation observed in Group 1 aligns with theories of embodied cognition (Waller et al., 2004; Chrastil and Warren, 2012), suggesting that direct environmental interaction leads to a more sequential encoding of spatial information. This also echoes Lynch’s (1960) seminal work on urban cognition, in which landmarks are considered key structuring elements of mental maps and spatial recall, especially in unfamiliar environments.

The use of navigation tools appears to modify this relationship. The spline curve for Group 2 also shows an upward slope, albeit less pronounced. The plateau is reached earlier, around 37 min of exploration, approximately halfway through, with the average exploration duration for Group 2 being 64 min. However, the downward slope, though present, is considerably less steep. This suggests that the impact of the exploration chronology on the drawing process is much less pronounced for individuals in Group 2.

Most distinctly, collective navigation showed a fundamentally different pattern. Group 3 displays an almost linear spline curve (*edf* = 1.844). The near absence of slope indicates a lack of any relationship between the duration to physically reach a place from the MSH (IV), and the duration for that place to appear on the corresponding sketch map (DV).

The differences observed between the three groups suggest that the navigation conditions significantly impacted (1) how participants acquired spatial knowledge during the exploration, and (2) how they transcribed this knowledge into sketch maps. Specifically, Group 1’s bell-shaped spline curve implies that these individuals developed a relatively detailed knowledge of the routes, and thereby landmarks, if referring to Siegel and White’s (1975) L-R-S theory. Direct experience in the Plaine Saint-Denis district likely resulted in procedural encoding in the form of a landmark-directions association, as initially suggested by Kuipers (1978) and more recently by Warren et al. (2019). Furthermore, this result highlights the critical role of the body movement in acquiring spatial knowledge (Waller et al., 2004; Ruddle et al., 2011; Chrastil and Warren, 2012), as well as the importance of path integration in navigation and wayfinding without cartographic aids (Etienne and Jeffery, 2004). However, our measurements do not allow for assessing the accuracy of the acquired spatial knowledge (estimations of directions, distances, etc.), thus not definitively confirming the presence or absence of survey knowledge encoding.

For map users, our results suggest a hybrid form of spatial knowledge acquisition. Group 2’s spline curve suggests that these participants recalled the first part of their journey before locating places on the sketch map without a clear temporal relationship to their itinerary. On the one hand, the descending slope of this curve – present but less pronounced compared to Group 1 – suggests that the use of mobile maps contributed to acquiring a configurational understanding of the environment, aligning with studies by Munzer et al. (2006), Ishikawa et al. (2008), Willis et al. (2009), and Quesnot (2016). On the other hand, the rising slope of this curve indicates that route knowledge developed alongside survey knowledge. This result challenges once again the sequential nature of Siegel and White’s (1975) L-R-S theory, while supporting Montello’s (1998) alternative framework, and the more recent work by Chrastil (2013) as well as Kim and Bock (2020).

The collective navigation condition revealed a distinct cognitive process. Group 3 differs from the other two: the fitting of its spline curve suggests that individuals in this group did not chronologically remember their route while they were drawing their sketch maps. This result implies that the collective interactions impacted (1) the transcription of individual cognitive maps into sketch maps, and potentially, (2) the encoding of spatial knowledge during exploration. The appearance of photos as pop-ups and the continuous display of subgroup members’ routes seem to alter, or even negate, the recall process of the followed route during sketch map making. In a previous study (Quesnot and Guelton, 2023), we hypothesized that collective interactions during the exploration of an unknown environment enhanced group cohesion, thereby mitigating collaborative inhibition (i.e., the decrease in a group’s mnemonic performance compared to that of its individual members) and improving the accuracy of collectively created sketch maps. However, we were unable to determine the impact of these interactions on the individual acquisition of spatial knowledge. Now, the comparison of the spline curves of the three study groups suggests that this is indeed the case. While individuals in Group 3 likely acquired route and landmark knowledge, the increased focus on the mobile map through real-time dissemination of geographic information (participants’ routes and geolocated photos) probably fostered the development of survey-type knowledge. In any case, this latest result converges on the idea that wayfinding is far from being an asocial activity (Dalton et al., 2019), and that the concept of “altercentric cognition” also applies to spatial memory (Kampis and Southgate, 2020).

These findings have important implications for both our theoretical understanding of spatial cognition and practical applications in navigation design. They suggest that the process of externalizing spatial knowledge through sketch maps is not uniform across different navigation conditions. This challenges the notion of a single, universal cognitive map and supports more nuanced models of spatial knowledge representation (Chrastil and Warren, 2014). However, it is important to acknowledge the limitations of our study. While our models reveal significant patterns, they only explain a small part of the dataset variability. Yet, this is not unusual in human and social sciences, where achieving a high explanatory percentage remains challenging due to the intrinsic complexity of human behaviours and interactions. In this study, the in-situ nature of the exploration adds an additional layer of complexity with a significant number of uncontrollable variables. Environmental and contextual factors, along with the diversity of individual responses, significantly contribute to the observed variability and obviously limit the models’ capacity to capture the full range of dynamics at play.

We believe that some intermediate variables could have influenced our results. First, “culture” plays a central role in the formation of cognitive maps (Heft, 2013). Yet, our sample partly consisted of foreign nationals (i.e., non-French) because of the main recruitment conditions regarding the unfamiliarity with the Plaine Saint-Denis district. Second, the visibility of landmarks appears equally crucial. Quesnot and Roche (2015) demonstrated that individuals exploring an unfamiliar environment were much more sensitive to the visual salience of places. Given that the exploration was entirely free and unsupervised by an experimenter, it was impossible to calculate each participant’s field of vision and accordingly measure the visual salience of each encountered place. Third, we must mention a bias related to the methodology we employed: to simplify the organization of the experiment, we met participants directly at the MSH. It is highly probable that most of them arrived by metro, thus walking up Avenue Georges Sand for nearly a hundred metres to reach the MSH. The locations encountered during this short travel may have been memorized albeit not visited during the *in situ* exploration itself. Fourth, we did not include walking speed or drawing speed as covariates in our statistical models. These behavioural variables may have influenced both the spatial extent of exploration and the timing of map creation. For instance, faster walkers may have encountered more locations without necessarily encoding them deeply, while slower or more deliberate sketchers may appear less efficient in terms of drawing time despite possessing accurate spatial knowledge. While we chose not to control for these dimensions to maintain model simplicity and focus on broader group patterns, future studies could integrate such measures for a finer-grained understanding of how exploration dynamics interact with spatial knowledge externalization.

## 7 Conclusion

This exploratory study advances our understanding of how different navigation conditions influence spatial knowledge acquisition and representation. The findings suggest a causal link between the chronological order in which individuals navigate an unfamiliar environment, and the way they design their sketch map. However, this explanatory relationship exhibits significant variations depending on the navigation conditions under examination. More precisely, our analyses using GAMM models with thin plate spline curves show that participants who directly experienced the environment (Group 1) tended to draw places in a chronological order that matches their exploration’s temporality. The use of a map seems to mitigate this phenomenon: individuals in Group 2 tended to follow a similar approach for the first half of their travel, then drew places sporadically without a clear relation to the chronology of their exploration. Collective navigation, enhanced by the interactive features of the shared mapping application, appears to nullify the impact of route chronological recall on the individual process of sketch map making. Indeed, the appearance order of places on Group 3’s sketch maps does not correspond to the order in which they were visited. In the end, these findings lead to a dual hypothesis that should be tested in a more extensive experiment, namely: during individual and direct exploration, people tend to make their sketch maps following a chronological order that reflects the route they followed earlier. Conversely, the interaction with cartographic tools and/or the collective navigation context promotes the externalization of spatial knowledge that is (1) less dependent on the exploration’s chronology, and (2) more oriented towards a configurational encoding of the environment.

## Data Availability

The raw data supporting the conclusions of this article will be made available by the authors, without undue reservation.

## Footnotes

^1^To conduct this experiment, approval by an ethics committee was not required, neither by the university of the first author nor by that of the second.
